# A Novel and Fast Purification Method for Nucleoside Transporters

**DOI:** 10.3389/fmolb.2016.00023

**Published:** 2016-06-09

**Authors:** Zhenyu Hao, Maren Thomsen, Vincent L. G. Postis, Amelia Lesiuk, David Sharples, Yingying Wang, Mark Bartlam, Adrian Goldman

**Affiliations:** ^1^Key Laboratory of Pollution Processes and Environmental Criteria, Ministry of Education, College of Environmental Science and Engineering, Nankai UniversityTianjin, China; ^2^Faculty of Biological Sciences, Astbury Centre for Structural Molecular Biology, School of Biomedical Sciences, University of LeedsLeeds, UK; ^3^Biomedicine Research Group, Faculty of Health and Social Sciences, Leeds Beckett UniversityLeeds, UK; ^4^Department of Molecular Biology and Biochemistry, College of Life Sciences, Nankai UniversityTianjin, China; ^5^State Key Laboratory of Medicinal Chemical Biology, Nankai UniversityTianjin, China; ^6^Division of Biochemistry, Department of Biosciences, University of HelsinkiHelsinki, Finland

**Keywords:** nucleoside transporters, membrane protein, vector construction, purification, expression

## Abstract

Nucleoside transporters (NTs) play critical biological roles in humans, and to understand the molecular mechanism of nucleoside transport requires high-resolution structural information. However, the main bottleneck for structural analysis of NTs is the production of pure, stable, and high quality native protein for crystallization trials. Here we report a novel membrane protein expression and purification strategy, including construction of a high-yield membrane protein expression vector, and a new and fast purification protocol for NTs. The advantages of this strategy are the improved time efficiency, leading to high quality, active, stable membrane proteins, and the efficient use of reagents and consumables. Our strategy might serve as a useful point of reference for investigating NTs and other membrane proteins by clarifying the technical points of vector construction and improvements of membrane protein expression and purification.

## Introduction

Membrane proteins represent 20–30% of all genes in most sequenced genomes (Krogh et al., 2001) and are targets for about half of all modern therapeutic drugs (Krogh et al., 2001; Bayley, 2009; Andreeva et al., 2014). Membrane proteins, such as membrane transporters, receptors, enzymes, and cell adhesion molecules, have been studied for decades on account of their critical biochemical roles in the maintenance of vital cellular functions (Bowie, 2005; Von Heijne, 2006; Almén et al., 2009; Anson, 2009). However, production of native membrane proteins has always proven to be a bottleneck in structural and functional studies (Lacapere et al., 2007; Midgett and Madden, 2007). Current production methods generally lead to a low yield of expression or result in overexpression of misfolded protein (Drew et al., 2008).

Our particular interest is in nucleoside transporters (NTs). Their study is biologically and clinically relevant because they are important in the uptake of nucleoside drug analogs. Furthermore, nucleosides, adenosine in particular, regulate many aspects of heart, brain, and immune system physiology (Johnson et al., 2012; Young et al., 2013; Rehan and Jaakola, 2015) and so have great potential for therapeutic applications in cardiovascular disease, inflammation, and cancer (Carrier et al., 2006; Hsu et al., 2012; Young et al., 2013; Valdés et al., 2014). However, the molecular basis of the functional mechanisms of nucleoside transport remain poorly defined. One of the main reasons lies in the difficulty of production and purification of recombinant NTs.

**Figure 1:**
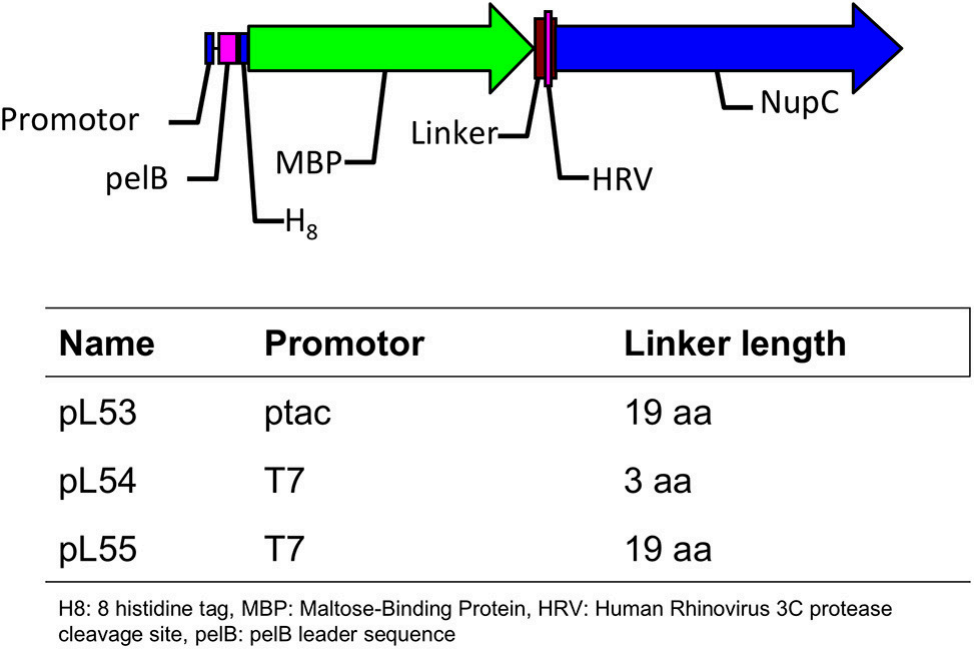
**Description of the plasmids used in this study**.

A standard approach for membrane protein production is the expression of the target membrane protein as a fusion protein, which can increase the stability and expression level of the target (Alexandrov et al., 2001; Heijbel et al., 2009) and/or makes it easier to follow the expression and purification (Drew et al., 2005). A diverse range of soluble proteins have been used as fusion partners including maltose binding protein (MBP), thioredoxin, green fluorescence protein (GFP), and glutathione S-transferase (GST) (Heijbel et al., 2009; Sammons and Gross, 2013; Gosch et al., 2014; Nguyen et al., 2014; Satoh et al., 2015). MBP is one of the most commonly used for the generation of fusion protein with membrane proteins in microbial expression systems (Lorenzo et al., 1997; Dälken et al., 2010; Xu et al., 2014). Because it is known to be well expressed and stable in the *Escherichia coli* periplasm, MBP is generally used as an N-terminal fusion of membrane proteins to be purified (Hu et al., 2011), but is not used as a purification tag because of the maltoside detergents used in solubilisation interfere. The inconvenience of using a His-tag directly as an N-terminal fusion of a membrane protein exposing its N-terminus in the periplasm is that the presence of the His_8_ positive tag can alter the protein topology (Von Heijne, 2006). To avoid this problem, we have used MBP as a linker between the purification tag (8xHis) and the membrane protein of interest, hence keeping the topological stability of the target as well as the flexibility of His-tag purification procedures (Ma et al., 2015). In addition, a specific proteolytic cleavage site is added between the MBP and the target protein. Classical approaches use proteases in solution together with the protein so, after cleavage, an additional step is needed to repurify the target protein from the protease and the cut tag. The approach we have undertaken in this study is to combine the use of a His tagged protease added directly onto the protein bound to the purification column. As a result, the target protein is specifically released into the flowthrough while the His-protease and the His-MBP are retained on the IMAC (immobilized metal-affinity chromatography) column, hence avoiding another round of purification. We have used NupC (*Escherichia coli*, Uniprot accession no.: P0AFF2) as a model protein to demonstrate the efficiency of this approach, while additionally optimizing and simplifying each prior purification step to decrease purification time. This optimized purification procedure has been successfully applied to other NTs (SI-Figure 1). In addition, we developed an improved expression system by changing the promoter and the linker described in our earlier study (Ma et al., 2015). Compared to our previous approach, the optimized method is faster and more cost effective. Similar optimization approaches can be generally applied to the production of other membrane proteins.

## Materials and methods

### Reagents and plasmids

We produced HRV-3C protease with an N-terminal octahistidine tag, which had been ligated into a pET28 derivative vector. The protease was purified by IMAC after expression in *E. coli* (Postis, unpublished). The detergents dodecyl-β-D-maltoside (DDM) and decyl-β-D-maltoside (DM) were from GLYCON Biochemicals GmbH (Luckenwalde, Germany), lauryldimethylamine-N-oxide (LDAO), and octaethylene glycol monododecyl ether (C12E8) were from Anatrace (Maumee, USA) and octyl β-D-glucopyranoside (OG) was from Affymetrix Inc (Santa Clara, USA). Isopropyl β-D-1-thiogalactopyranoside (IPTG) was from Melford Ltd. (Chelsworth, UK). The vectors pL53 and pL55 employed in this study are as described in our previous report (Ma et al., 2015), and the same strategy was used to generate pL54.

### Protein expression

The optimal expression conditions of proteins with the different vectors were obtained from expression optimizations following protocols in (Baldwin, 2000). A single colony was transferred in 300 ml Luria Broth medium for incubation overnight at 37°C. The large scale expression of the target protein was conducted in 30 l fermentor (Infors HT). The cells were grown in Terrific Broth medium at 37°C until an OD_600_ of 0.6 was reached. The expression of the target of interest was induced by the addition of 0.5 mM IPTG. The cells were harvested after 6 h by centrifugation.

### Membrane preparation

Membrane preparation was performed as previously published (Ma et al., 2015). Briefly, after being resuspended at 6 mg/ml (wet cell weight) in phosphate-buffered saline (PBS), the cells were disrupted at 30 kpsi using a TS series cell disruptor (Constant Systems Ltd., UK). The cell debris were removed by centrifugation at 14,000 g for 45 min. The membrane fraction was pelleted by ultracentrifugation at 100,000 g for 2 h. The membrane pellet was washed twice with PBS buffer. The final membrane pellet was resuspended in PBS buffer to a concentration of total membrane protein between 20 and 40 mg/ml and snap-frozen in liquid nitrogen.

### Solubilisation trials

Solubilisation trials were carried out using a final membrane protein concentration of 5 mg/ml in solubilisation buffer (Table 1) and 1% (w/v) of the tested detergents. Five different detergents were tested: DDM, DM, LDAO, C12E8, and OG. The resuspended samples were gently mixed for 2 h at 4°C. The suspensions were centrifuged at 100,000 g for 1 h. The protein of interest in the collected supernatants was detected by western blot (anti-histidine tag antibody) and its amount compared to the total amount of NupC present before centrifugation.

**Table 1:** **Buffers**.

**Buffer**	**Composition**
1*PBS	10 mM Na_2_HPO_4_, 1.8 mM KH_2_PO_4_, 137 mM sodium chloride, 4 mM potassium chloride, pH 7.4
Solubilisation buffer	50 mM Tris pH 7.4, 150 mM sodium chloride, 5% (w/v) glycerol, 5 mM imidazole
Purification buffer	50 mM potassium phosphate buffer pH 7.4, 150 mM sodium chloride, 5 mM imidazole, 5% glycerol (w/v), and 1% DDM
Wash buffer	50 mM potassium phosphate buffer pH 7.4, 150 mM sodium chloride, 15 mM imidazole, 0.05% DDM, and 10% glycerol (w/v)
Cleavage buffer	50 mM potassium phosphate buffer pH 7.4, 150 mM sodium chloride, 5% glycerol (w/v), and 0.05% DDM

### Protein purification

All experiments were performed at 4°C unless stated otherwise. The standard purification was described in Ma et al. (2015) and the optimized purification was carried out as follow. Membrane proteins (100 mg) were solubilised in purification buffer for 1 h. The non-solubilised material was removed by centrifugation (100,000 g for 1 h). The supernatant was collected and incubated with 4 ml HisPur^TM^ Cobalt resin (Thermo Scientific^TM^) for 1 h on a roller mixer. The resin was then packed into a column and washed with 10 column volumes (CV) of wash buffer. The washed resin was then resuspended in 1 CV of cleavage buffer and incubated for 2 h with the appropriate amount of HRV-3C protease (molar ratio target protein to protease 1: 5). Afterwards, the flowthrough containing the protein of interest was collected and the resin was washed with 1 CV of cleavage buffer. Excess protease was removed from the protein solution by incubating it for 15 min with 4 ml of Ni-NTA resin slurry (Thermo Scientific^TM^) on a roller mixer. The cleaved protein was collected by transferring the suspension to an empty column and washing the resin with 1 CV of cleavage buffer. The protein was concentrated with a 30 kDa cut-off concentrator (Sartorius Stedim Biotech, 20 mL) for further experiments.

## Results

### Advantages of the novel pL55 MBP fusion vector

**Figure 2:**
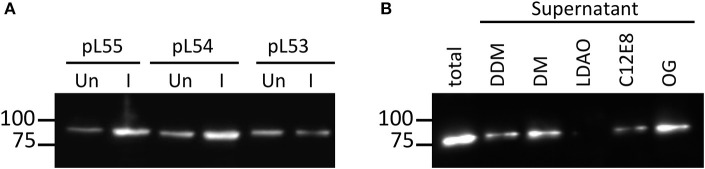
**Expression level and solubilisation level of NupC expression. (A)** Comparison of the expression of NupC in pL53, pL54, pL55. Un, uninduced cell culture; I, cell culture after induction; **(B)** Solubilization trial of NupC expressed in the pL55 vector using five different detergents.

**Figure 3:**
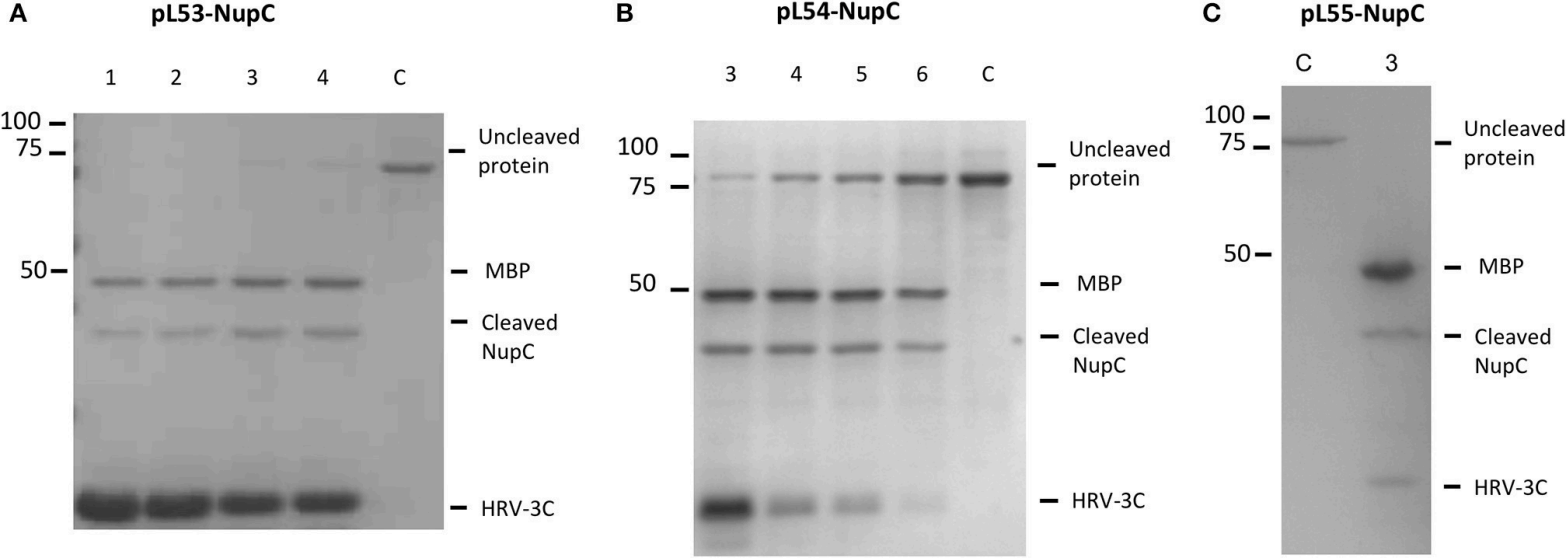
**Cleavage efficiency of NupC in the presence of different molar ratios of NupC:HRV3C after 16 h of digestion. (A)** pL53 construct, **(B)** pL54 construct, and **(C)** pL55 construct. 1, 1: 10 ratio; 2, 1: 5 ratio; 3, 1: 2 ratio; 4, 1: 1 ratio; 5, 2: 1 ratio; 6, 5: 1 ratio; C, control NupC without HRV-3C.

The expression of NupC is under the control of a strong promoter (T7) in pL54 and a weak one (ptac) in pL53. On the other hand, the linker between the MBP and HRV3C protease cleavage site is shorter in pL54 than in pL53 (Figure 1). The stronger promoter led to a higher level of expression (Figure 2A). Furthermore, as shown in Figure 3, the longer linker present in the protein expressed by pL53 led to better cleavage of the tag. Less HRV-3C protease is required to completely cleave protein expressed in pL53 than in pL54.

We therefore created a new MBP fusion vector, pL55, which includes a T7 promoter, a periplasmic targeting sequence (pelB), an octa histidine tag, MBP, and a human rhinovirus 3C protease cleavage site (HRV3C). The level of expression of NupC in a pL55 context is almost equivalent to level observed for pL54, and approximately double compared to pL53 (Figure 2A). This suggests that constructs with a T7 promoter (pL54 and pL55) could yield much more target membrane protein than constructs with a ptac promoter (pL53).

The efficient solubilisation of NupC produced by the pL55 construct by 5 different detergents shows that changing the length of the linkers or the strength of the promoter does not lead to the expression of the recombinant protein into inclusion bodies (Figure 2B). The pL55 vector, with a strong promoter and a long linker, thus combines the advantages of both pL53 and pL54 vectors. The expressed NupC was functional in all three different vectors according to a transport assay (Ma et al., 2015).

Additionally, we tested the cleavage efficiency of NupC expressed in pL53, pL54, and pL55 in the presence of different molar ratios of NupC:HRV3C after 16 h (Figure 3). The results show that the cleavage efficiency of NupC in pL53 was clearly higher than in pL54. The fusion NupC in pL55 can be cleaved efficiently following the same molar ratio NupC:HRV of 5:1 obtained from the test in pL53, as they possess equivalent length linker.

### Optimization of purification using MBP fusion protein

A crucial parameter in membrane protein purification is the time required for their purification. The low stability of most membrane proteins inevitably leads to decreased protein yields due to denaturation, aggregation, and proteolysis during long purification protocols.

**Figure 4:**
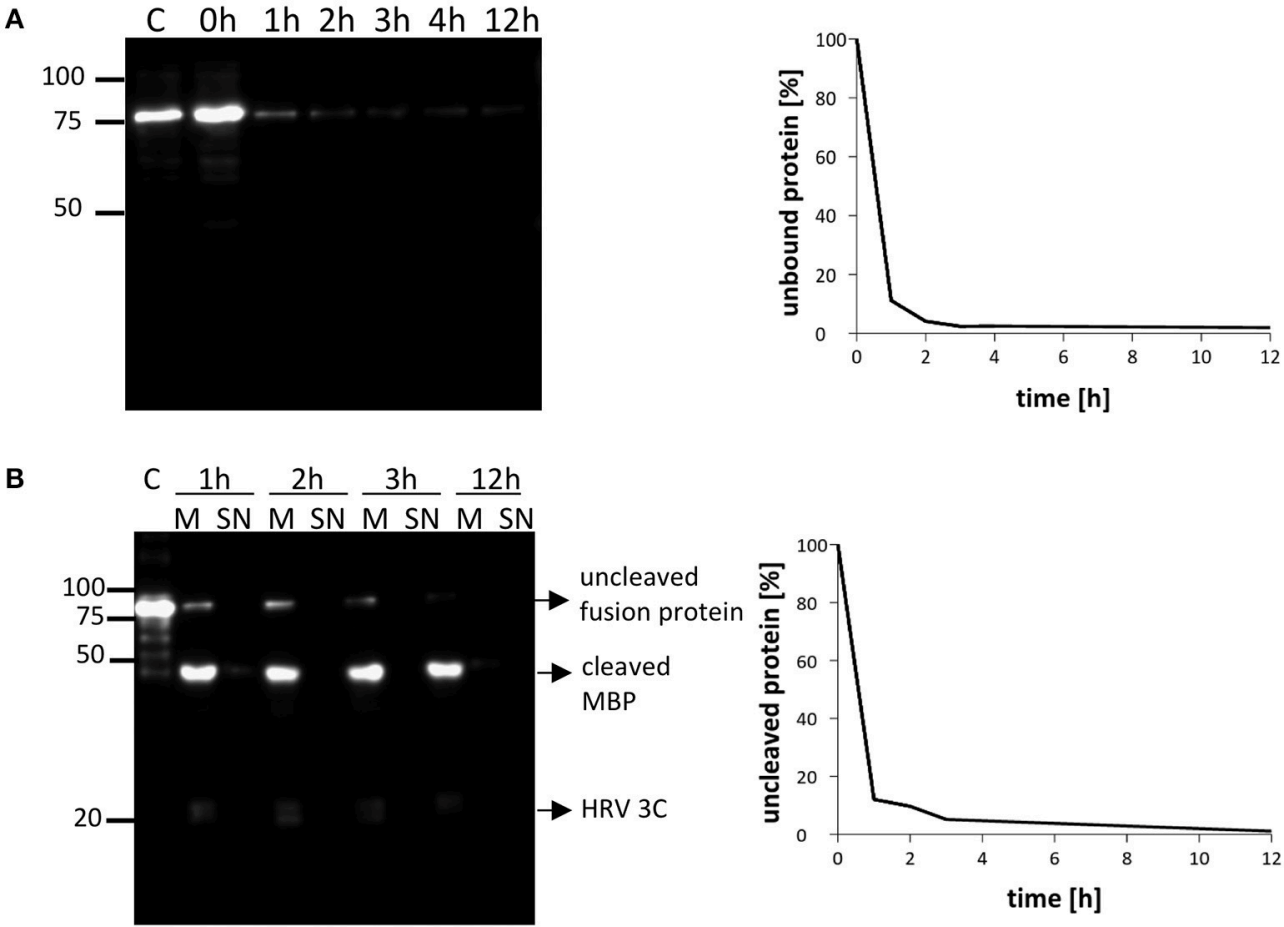
**Time course of NupC (pL55) binding to the resin and cleavage of the resin (molar ratio NupC:HRV3C of 5:1). (A)** Binding rate of NupC, the presence of the protein in the supernatant is detected by western blot using an antibody against the His tag. **(B)** Rate of cleavage of the protein from the cobalt resin. The quantity of uncleaved protein is measured by western blot using an antibody against the His tag. M, incubation of resin mixture after short spin (Cobalt resin and 1st dialysis buffer, plus HRV-3C); SN, supernatant of mixture after short spin (Cobalt resin and 1st dialysis buffer, plus HRV-3C); C, control fusion NupC.

Our previous purification protocols for NupC required long binding times (usually overnight) of the resin with solubilized membrane proteins for complete adsorption, in order to maximize the final yield. In this new approach, we decided to investigate how the amount of unbound NupC varied with incubation time. The Co-resin was incubated with solubilized NupC for 12 h. Samples were taken at 1, 2, 3, 4, and 12 h, centrifuged, and the amount of unbound target protein in the supernatant was determined via western blot and compared to the total initial amount. We found that more than 95% of the solubilized tagged protein was bound to the resin within 2 h, suggesting that the binding time could be significantly shortened from overnight to 2 h to avoid loss of activity during the long purification process (Figure 4A).

Conventional membrane protein production methods also generally require a long time for cleaving the MBP fusion protein off (overnight or even longer). To investigate how quickly cleavage occurs, we added the protease to the column-bound membrane protein (molar ratio NupC:HRV of 5:1) and compared the cleaved to uncleaved protein ratio at different incubation times by western blot (anti-histidine tag antibody) (Figure 4B). The results indicate that about 90% of the fusion protein was cleaved within 2 h, and that a longer incubation time did not significantly increase the cleavage yield. Therefore, the digestion time was reduced from 16 to 2 h in the optimized method. To determine the amount of protease required to achieve this, we examined the cleavage efficiency using different amounts of protease against recombinant NupC in the three different vectors (Figure 3). Our findings show that a 5:1 molar ratio of NupC:HRV protease was sufficient during the 16 h cleavage of NupC in the pL53 and pL55 vectors, which possess the same long linker. On the contrary, cleavage of NupC in pL54 with a short linker proved difficult, even using a large amount of protease (NupC:HRV of 1:1).

**Figure 5:**
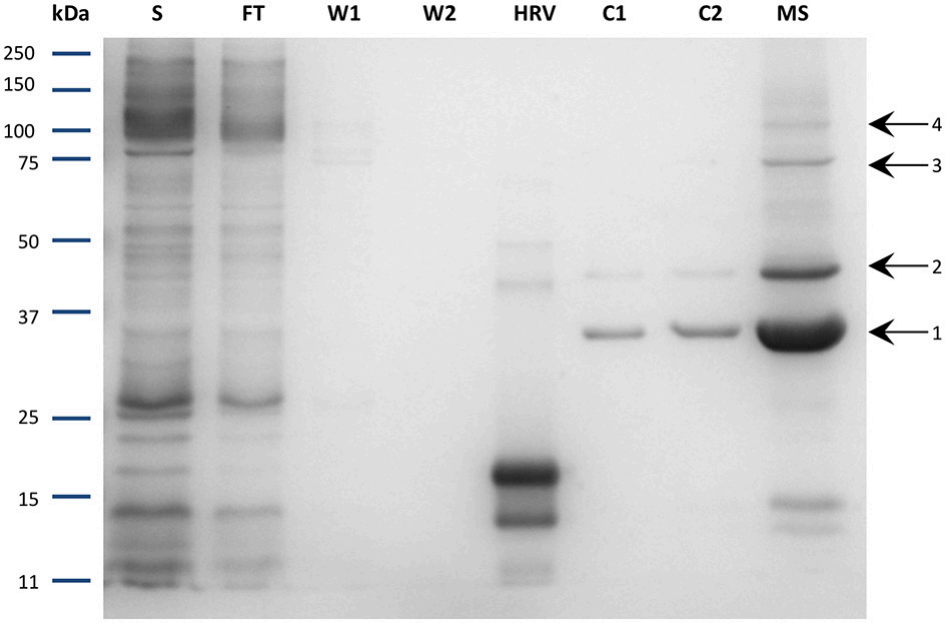
**SDS-PAGE of the purified NupC in DDM performed via the optimized procedure**. S, solubilized fraction after ultracentrifugation; FT, Flow through of cobalt resin; W1, washing step 1; W2, washing step 2; HRV, purified human rhinovirus 3C-protease; C1, fractions after cleavage; C2, flow through after incubation with Nickel resin to remove residual protease; MS, concentrated protein sample with labeled bands investigated via mass spectrometry. Band 1: expected molecular mass of NupC monomer.

The purity of the protein was subsequently evaluated. After cleavage and concentration steps, the purified protein was loaded onto SDS-PAGE and stained with Coomassie Blue. Unexpectedly, three main bands were observed on the gel in addition to the NupC monomer (Figure 5). All four bands were identified as NupC by mass spectrometry analysis (data not shown), consistent with the fact that NupC can form higher oligomers and in particular trimers as seen in the 3D structure (Johnson et al., 2012). This suggests that purified NupC retains its native trimeric structure even in SDS-PAGE. Band 1 (less than 37 kDa) is presumably the NupC monomer. Bands 2 and 3 correspond to a dimer and trimer respectively, while band 4 (above 100 kDa) is some higher-order oligomeric structure.

This purification strategy has broad range applications for other membrane proteins. This is illustrated by the successful purification of the nucleoside transporter from *Anoxybacillus flavithermus, Bacillus halodurans, and Rhodothermus marinus* using this optimized strategy (SI-Figure 1).

## Discussion

For a long time, the low yield of membrane proteins has presented a major obstacle for their structural and functional studies, especially for NTs. Many attempts have been made to overcome this problem using various expression strains, vectors and media for membrane proteins (Winstone et al., 2002; Drew et al., 2006; Öberg et al., 2011; Schlegel et al., 2012). The MBP protein, which was reported to increase the stability and solubility of membrane proteins (Gruswitz et al., 2005; Raran-Kurussi et al., 2015), was introduced into our nucleoside transporter expression vectors. Meanwhile, it has been reported that different promoters could change the expression level of target proteins, and a strong promoter, such as the T7 promoter in pL54, can facilitate the overexpression of the target protein (Berg et al., 2012; Phan et al., 2012; Hensel et al., 2013; Binder et al., 2014). Through combining a strong promoter for enhanced protein expression with MBP enhancing the protein stability as fusion partner we were able to construct a new expression vector (pL55), which resulted in significantly higher protein yields than our earlier vectors.

**Figure 6:**
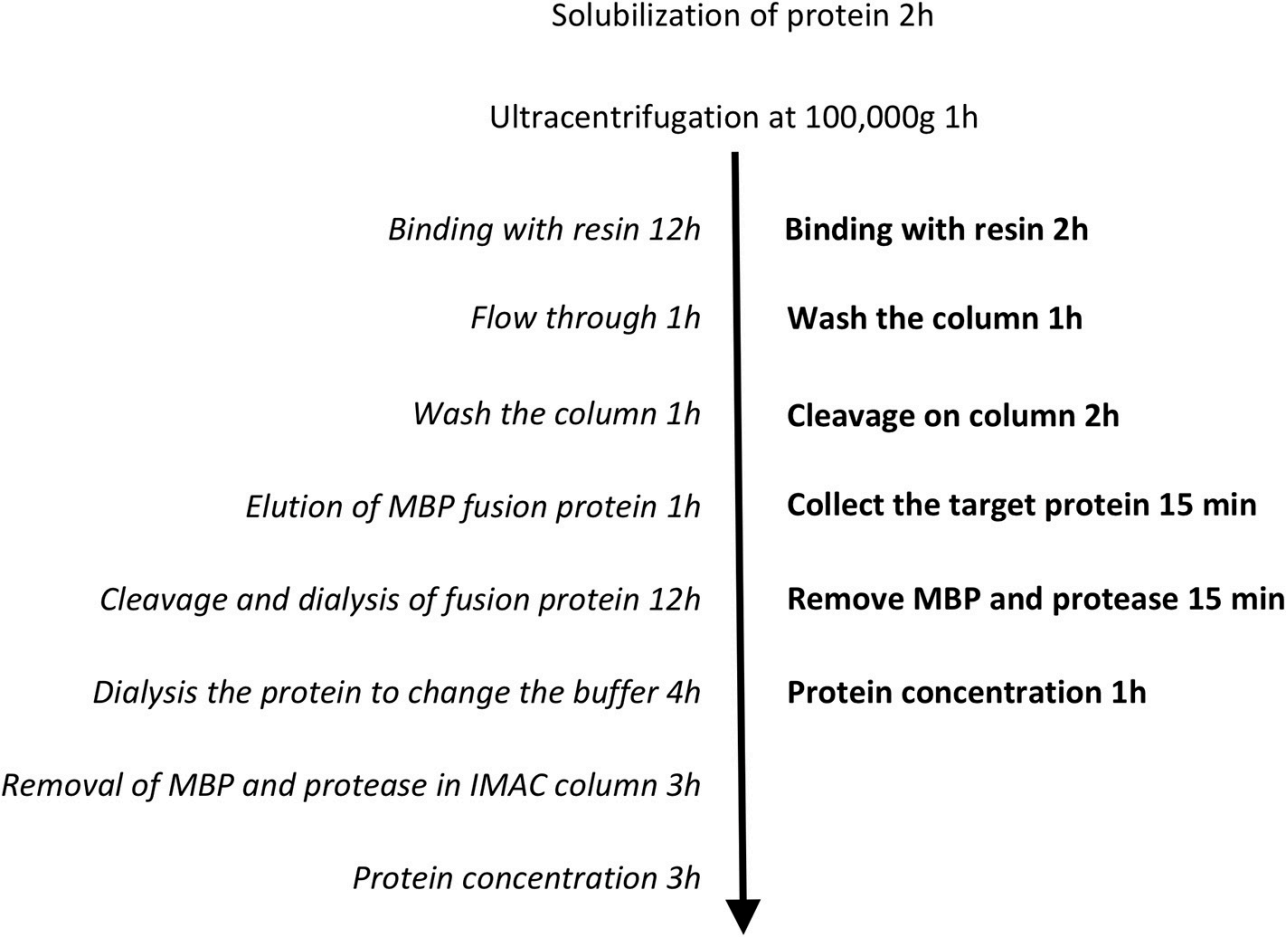
**Technical routes of the two purification protocols**. The conventional method is shown in italics, and the optimized one in bold.

A limiting step in the purification of MBP fusion proteins has often been the rate of proteolytic cleavage of the MBP from the target proteins. It was reported that the incomplete digestion resulted from the presence of certain detergents during purification (Mohanty et al., 2003). Hu and colleagues speculated that detergent solubilisation of the hydrophobic cleft on the surface of MBP may form a binding site for the hydrophobic domain of the target membrane protein, which potentially results in a stable non-covalent complex following protein cleavage (Hu et al., 2011). In other words, the activity of the proteolytic enzyme can be dramatically reduced in the detergents used to solubilize membrane proteins (Vergis and Wiener, 2011). However, our results suggest that the rate of digestion is also dependent on the length of the linker between MBP and the target protein. We observed that digestion was more efficient when linkers were longer (pL55 and pL53, both 19 amino acids), as opposed to a shorter linker (pL54, 3 amino acids) (Figure 3). This is presumably because the cleavage site is more exposed and therefore more accessible to proteolytic enzymes when long linkers are employed. Indeed, smaller amounts of protease were needed to perform efficient digestion on the new His-MBP-NupC fusion expressed from our new vector, pL55, compared to pL54, a similar construct with a shorter linker. This means that the target protein can be purified much faster using the new vectors and methods (Figure 6): the time taken after ultracentrifugation is reduced from 37 to 6.5 h—almost a factor of six. Additionally, compared to the traditional purification protocol, the new method significantly reduces the consumption of reagents and lab consumables.

Furthermore, our new optimized method is particularly interesting for crystallization purposes. In traditional purification experiments, large volumes are used to elute the target protein efficiently. This implies that the purified protein is very diluted and a concentration step is required before downstream application. This in turn concentrates the detergent and has an impact on crystallization efficiency and crystal quality (Prince and Jia, 2013). Our optimized protocol describes how adding the protease directly onto the column-bound target protein reduces the volume of elution, hence yielding a more concentrated protein. Further protein concentration steps are therefore minimized, avoiding unnecessary collateral increase in detergent concentration, which is an important asset in crystallographic studies. Our new optimized strategy presents strong advantages over classical approaches in terms of time, cost, and quality of membrane protein purification for crystallographic studies.

## Author contributions

AG, ZH, VP, YW, MB designed the experiments. ZH, VP, AL, MT, DS performed the experiments. The data were analyzed by ZH, MT, VP, AL, AG. The paper was written by ZH, MT, VP, YW, MB, and AG.

## Funding

The authors are grateful to the financial support from the National key fundamental research project (973 project, 2014CB560709, to MB), National Science Foundation of China (31570128 to MB and 31270545 to YW), and the Royal Society Wolfson Research Merit Award, the Wellcome Trust (ref. 019322/7/10/Z), BBSRC (BB/M021610/1) and the Academy of Finland (1286429) (all to AG). ZH was funded by an award from the China Scholarship Council.

### Conflict of interest statement

The authors declare that the research was conducted in the absence of any commercial or financial relationships that could be construed as a potential conflict of interest.

## Supplementary material

SI-Figure 1 **SDS-PAGE of the purified CNT from ***Anoxybacillus flavithermus (Af), Bacillus halodurans (Bh), Rhodothermus marinus (Rm)*** in DDM performed via the optimized procedure**.

## Abbreviations

C12E8: octaethylene glycol monododecyl ether
DDM: dodecyl-*β*-D-maltoside
DM: decyl-*β*-D-maltoside
GFP: green fluorescence protein
GST: glutathione S-transferase
IMAC: immobilized metal-affinity chromatography
IPTG: isopropyl *β*-D-1-thiogalactopyranoside
LDAO: lauryldimethylamine-N-oxide
MBP: maltose binding protein
NT: nucleoside transporters
OG: octyl *β*-D-glucopyranoside.
