# Supplementary figures and images for: A Novel and Fast Purification Method for Nucleoside Transporters
Source: Front Mol Biosci. 2016 Jun 9;3:23. doi: 10.3389/fmolb.2016.00023 (PMC4899457; doi:10.3389/fmolb.2016.00023)

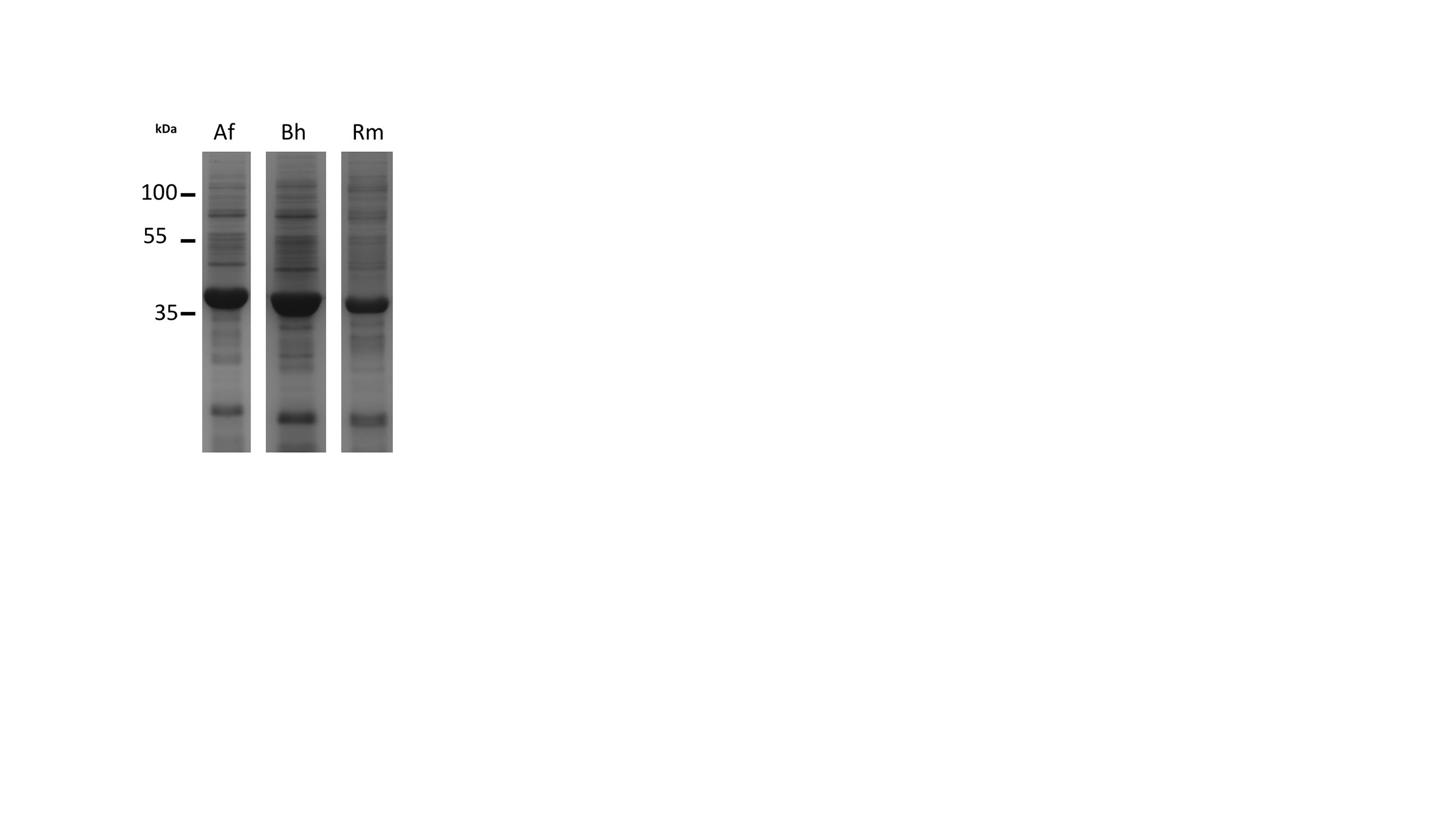

Supplement: SI-Figure 1 — SDS-PAGE of the purified CNT from Anoxybacillus flavithermus (Af), Bacillus halodurans (Bh), Rhodothermus marinus (Rm) in DDM performed via the optimized procedure. [file Image1.tiff]
